# Health systems readiness for adopting mhealth interventions for addressing non-communicable diseases in low- and middle-income countries: a current debate

**DOI:** 10.1080/16549716.2018.1496887

**Published:** 2018-07-24

**Authors:** Anam Feroz, Muhammad Masood Kadir, Sarah Saleem

**Affiliations:** Department of Community Health Sciences, The Aga Khan University, Karachi, Pakistan

**Keywords:** Health system preparedness, mobile phone-based health interventions, non-communicable diseases, low and middle-income countries, current debate

## Abstract

In low-and-middle-income countries, epidemiologic transition is taking place very rapidly from communicable diseases to non-communicable diseases. NCDs mortality rates are increasing faster and nearly 80% of NCDs deaths occur in LMICs. Existing weak health systems of LMICs are undergoing a devastating human and economic toll as a result of increasing treatment costs and losses to productivity from NCDs. At the same time, the increasing penetration of mobile phone technology and the spread of cellular network and infrastructure have led to the introduction of the mHealth field. While mHealth field offers a great promise to prevent and control non-communicable diseases in low-and-middle-income countries: there is a great debate going on to explore health systems readiness for adopting mHealth technology to address NCDs in LMICs. There are a number of factors which determine health systems readiness and response for adoption of mHealth technology including preparedness of healthcare institutions, availability of the resources, willingness of healthcare providers and communities. We have discussed these factors to understand health systems preparedness to adopt mHealth field for prevention and control of NCDs. To adequately integrate mobile-phone-based health interventions into existing health systems, these factors should be dealt up-front through constant effort to improve health systems response for NCDs. Currently, there is insufficient empirical and policy evidence on this research area and therefore future research and policy dialogue should be directed to assess the health systems willingness for mHealth adoption principally to address NCDs in the context of LMICs.

## Background

Recently, low- and middle-income countries (LMICs) are undergoing an epidemiologic transition from infectious diseases to non-communicable diseases (NCDs) [1,2]. NCDs have now become the leading cause of death – they have been the world’s number one silent killer for too long and have affected the “bottom billion and G20 countries alike’’[3]. NCDs cause an estimated 36 million deaths each year, including 9 million people dying before the age of 60 years. Nearly 80% of NCD deaths occur in LMICs [4]. The estimated cumulative lost economic output associated with NCDs is around US$7 trillion over the period 2011–2025 through health-care costs and productivity losses [5]. Four types of NCDs including cardiovascular diseases, chronic respiratory diseases, diabetes and cancers make the major contribution to overall morbidity and mortality in LMICs [5]. World Health Organization Action Plan on NCDs, the UN General Assembly Resolution on NCDs, and the Global Alliance for Chronic Diseases (GACD) provide strong evidence-base that increasing attention is being given to the impact of NCDs on health and development [6]. In Millennium Development Goals (MDGs), no clear commitment was made to address NCDs. For the first time, the United Nations Summit on 2030 agenda for Sustainable Development recognized NCDs as a major challenge for sustainable development and the issue of NCD is brought on the global development agenda [7].

Despite NCDs being largely preventable, the global disease burden from NCDs is predicted to progress over time. Existing weak health systems, especially those in LMICs, will undergo a devastating human and economic toll as a result of increasing treatment costs and losses to productivity from NCDs [8,9]. If action is not taken now to prevent and control NCDs, the burden and corresponding costs will grow substantially, which will place great stress on health systems. The impending global threat of NCDs, combined with weak health systems, calls for urgent solutions that have wide reach, strong potential for scale-up and have the ability to strengthen existing health systems [8].

According to the International Telecommunication Union’s (ITU) 2016 report, the number of mobile phone subscriptions has reached to 5 billion people worldwide, and this figure is expected to grow and surpass the world population in the years ahead. Worldwide, 95% of population reside in an area which is covered by a mobile-cellular network; and 84% of the people has access to mobile broadband networks [10]. This recent explosion and near ubiquity of mobile phone uptake around the world may offer opportunities for NCD prevention, health promotion, treatment and disease management. mHealth efforts focused on NCDs are already being implemented, and new evidence based on rigorous trials have begun reporting the benefits of text messaging, automated telephone monitoring, treatment reminders and self-care support for improving health outcomes related to chronic disease management [10–12].

The adoption of mHealth technology in high-income countries is over 60% compared to 20% among upper-middle, lower-middle and low-income countries [13]. This significant difference is likely associated with better awareness and understanding of mHealth technology by the health systems of the high-income countries where country’s economic growth corresponds to technological advances. Due to better understanding of the mHealth field and availability of the funds, health systems of the high-income countries show readiness for the adoption of mHealth solutions which is fundamental for reducing disease burden of NCDs [13].

In LMICs, most health systems are overstrained and continually challenged by the need to make complex decisions about competing priorities [14]. Since mHealth currently lacks a robust evidence base to substantiate its impact on preventing and treating NCDs, it is understandable that most LMICs health systems encounter conflicting priorities as their main barrier. Competing priorities generally means that health systems attention and funding is usually distributed to other interventions ahead of mHealth, or reflects a lack of awareness or understanding of the mHealth field [13].

The perceived benefits of mobile phone-based health interventions carry a great potential for prevention and control of non-communicable diseases in resource-constrained LMICs [15,16]. A wide range of mobile-based interventions exists for preventing and controlling NCDs. These include reminder text message for promoting healthy lifestyle in individuals at risk of developing NCDs, treatment reminders for patients suffering from NCDs, mHealth apps for self-care support for chronic disease management and automated monitoring using wireless and wearable sensors for diagnostic purposes [17]. Moreover, mobile interventions are also used for remote learning, particularly for improving healthcare workers knowledge and skills for effectively dealing with patients with long-terms illnesses [1,17]. Each of these mHealth interventions affect health systems of resource – constrained LMICs in many different ways. Some mHealth interventions reduce costs to the health system; other interventions tend to improve access to healthcare services, while few interventions focus on improving the quality and effectiveness of the healthcare services [18]. However, in order to gain the benefits of mHealth technology in LMIC, it is significant to explore the health systems readiness for adopting mobile phone-based health solutions to address NCDs in LMICs. Health systems readiness for adopting mHealth can be defined as the preparedness of healthcare institutions, availability of the resources, willingness of healthcare providers and communities for accepting the change brought by the technology. Healthcare systems may fail to successfully adopt mHealth as a result of lack of readiness among the organizations, institutions, healthcare providers and communities [19]. Exploring healthcare systems readiness is essential to successful adoption and implementation of mHealth solutions for addressing NCDs [20]. This debate is intended to stimulate awareness about the burden of NCDs in resource-constrained LMICs while looking at the readiness of the health systems for adopting mobile Health to control and prevent NCDs.

## Preparedness of healthcare organizations for mHealth adoption

In most LMICs, Government policies and healthcare institutions are not encouraging the use of mHealth and Information and Communication Technologies (ICTs) in healthcare sector to address NCDs and other illnesses. The lack of Government and organization interest to adopt mHealth and ICT has led to reduced attention and investment in setting up mHealth services for NCDs. The investments are usually made on other domains ahead of ICTs which eventually result in lack of infrastructure development for delivering mHealth services in LMICs [21]. The priority is given to other interventions and programs because of the inadequate human and financial resources and therefore most of the pilot mobile-based health interventions fail to achieve success and are not incorporated in existing healthcare programs [22]. The adoption of mHealth interventions at organizational level is limited by various other factors, including high operational cost of mHealth technology, maintenance cost of the mHealth infrastructure, poor internet connectivity in the remote areas, electricity shortages, and barrier of using local language in mHealth applications [21,23]. The limiting factors should be dealt up-front through constant effort to improve organizational capacity to adopt mHealth for addressing NCDs. The mHealth experts’ advocate the dissemination of the findings of existing mHealth programs for NCDs tested in different geographical locations, as it has a catalytic effect on health systems for mHealth adoption. In addition, mHealth practitioners encourage the engagement of healthcare organizations and institutions in the design phase of mHealth interventions [21].

## Healthcare provider readiness for mHealth adoption

The healthcare providers’ willingness for mHealth adoption to address NCDs is determined by various factors including appropriate use of mHealth technology in local context, capacity of health providers and front-line health workers, trust of providers on the potential of mHealth technology, use of relevant mHealth services to address NCD burden and acceptance for change in current job responsibilities [24]. The healthcare providers are motivated to use mHealth intervention when the innovation is appropriately used to address the needs of local population in order to decrease the NCD burden. Thus, the goals and ambitions of health care providers should be identified and the behaviors and interest of providers should be considered while initiating projects that involve ICT and mHealth [25]. The second important factor that determines providers’ willingness for mHealth adoption is the capacity of the healthcare provider to use mHealth innovation to control and treat NCDs [24,25]. Therefore, the capacity of healthcare provider should be assessed before introducing technology in healthcare settings, as mHealth services demand appropriate skills to practice new technology. The lack of provider’s capacity to practice mHealth services eventually affects the implementation and delivery of mHealth services [24,25]. At the same time, due to growing reliance on mobile phone technology and decreasing mobile phone costs, the smart phones usage has increased amongst the LMIC population. The increased use of smart phones for a variety of reasons in everyday lives give individuals transferable skills for engaging in actions that can help prevent and manage chronic diseases [26].The comprehensive training of health providers and front-line healthcare workers on mHealth services, including accurate knowledge about mobile device and intervention can improve their ability to use mobile-phone-based health applications for treatment and control of NCDs [21]. Another significant element that governs the adoption of mHealth solutions is the trust and confidence of healthcare providers and implementers on the proposed mHealth intervention. The disbelief on the technology by the providers affects the adoption, implementation and delivery of mHealth services. Thus, the trust of providers on technology should be considered as a prerequisite for the adoption and implementation of mHealth services for NCDs [24,25]. Furthermore, many providers do not adopt mHealth services because it’s either not available or the services which are offered are not pertinent to address the NCD issue. Thus, the relevance of using mHealth technology for treating NCDs should be ensured by the implementers and experts of mHealth services [24,25]. In addition, it is also significant to understand that how the introduction of new mHealth technology will impact the current job responsibilities of healthcare providers. It is advocated that healthcare providers are less likely to use and adopt mHealth technology if it does not integrate well with their existing job duties. Thus, mHealth implementers and experts should principally focus on integration of technology to the provider’s current job [24,25].

In LMICs, adoption of mHealth services by healthcare providers is limited by various factors including apprehensions about workload, remuneration and lack of supervision and training. In few cases, where healthcare providers are willing to use mHealth solutions for addressing NCDs, healthcare infrastructure is not adequately provided, which in turn affects health practioners’ motivation to integrate mHealth component in existing service delivery structure of NCDs. In various other low-resource settings, healthcare providers and front line health workers are using mHealth solutions to prevent and control NCDs especially in remote areas of the country. The healthcare providers find mHealth as an effective and efficient solution to treat NCDs as face-to-face consultations are minimized and are only conducted when needed. This eventually enhances the overall effectiveness of the physician’s workflow. The visual consultation allow providers to share NCD risk scores and treatment progress with patients which eventually improves health provider’s confidence in counseling and treating patients more efficiently [5]. Moreover, mHealth applications for NCDs help providers to make more knowledge- able and evidence-informed clinical decisions [21].

In resource constrained settings, health services are mainly delivered through community health workers (CHWs) with limited resources and trainings. These CHWs get overstrained as their work environment pose high demands on their time and efforts. mHealth offers solutions by capitalizing on these limited resources by introducing simple techniques, processes and tools that help improve work-flows [27].

## Community willingness to adopt mHealth

Healthcare providers and mHealth experts anticipate slow adoption and uptake of mHealth services by the community members because a new innovation is not adopted simply unless it is given due awareness and significance. The community readiness for mHealth adoption to address NCD burden is determined by various factors including demographic characteristics of the users, affordability of the mHealth services, and capacity of the users, relevant and appropriate use of mHealth services for the local communities, socio-cultural factors and trust of users on mHealth technology. The demographic characteristics of communities appear to affect the uptake and adoption of mHealth services for controlling NCDs. User’s ethnicity, age, marital status, and geographical location appears to influence access to, uptake of, and satisfaction with the mHealth services. Younger people has a faster uptake for mHealth services then older population, as younger people are more motivated to accept change and are keen to know health-related information and test new innovations [28]. Thus, demographic characteristics of communities’ should be considered during the process of mHealth adoption and implementation [28]. Another significant factor that determines mHealth adoption and uptake is the affordability of the mHealth services by the communities. It is important to know that the suggested new mHealth intervention for NCD control and prevention is affordable for the community or not, as lower socio-economic groups may not have personal mobile phone access and may have lower levels of home internet access with reduced ability to pay for mHealth services. Thus, economic status should be considered throughout the course of mHealth adoption and implementation as it appears to affect mHealth uptake for the control and prevention of NCDs [24,28]. Moreover, educational accomplishment also appears to affect adoption of mHealth services, as the use technology and internet demand appropriate IT skills from general populations. It has been found out that attaining a higher level of education and in turn receiving a higher annual income is positively co-related with higher use of information technology including mHealth [28]. Thus, higher education is correlated with the greater awareness and use of mHealth services. For that reason, mHealth implementers and experts should take in to account the capacity and educational attainment of communities during the course of mHealth implementation for prevention and control of NCDs [28]. In addition, communities demand relevant services and information provided through mHealth intervention for the reduction of NCDs. The language used in the mHealth interventions and applications should be easily understood by the target audience. Thus, the mHealth experts should principally focus on the content of mHealth services while delivering services to the communities [24,28]. Nevertheless, it is also significant to understand the limitations to the use of technology due to sex, race or other socio-cultural factors. The influence of the socio-cultural factors on vulnerable groups affects the uptake of mHealth services by communities’ of LMICs. The security and privacy concerns associated with the use of mHealth services appear to affect the uptake of mHealth interventions. Thus, socio-cultural factors should be considered during the design phase of mHealth services, as it is recognized as a major barrier for the uptake of mHealth interventions [28]. Last, it is significant to explore communities trust and confidence on the proposed new mHealth initiatives and their understanding about the implications of technology use. While technology can be a key driver of community development; it can also be a barrier due to anxiety and distrust among people. Therefore, it is important to use simple and well-tested mHealth intervention for NCDs so that communities can trust its utility [24,28].

## Conclusion

The notion of Universal Health Coverage (UHC) is integral to the achievement of Sustainable Development Goals (SDGs) 2030 agenda. The use of information and communication technologies (ICT) such as eHealth and mHealth are vital to achieve UHC [29]. The recent penetration of mobile phone subscriptions has resulted in increased adoption of mHealth services for NCDs control and treatment in high-income countries. Although in LMICs the field of mHealth is still in its infancy, it can emerge as a vital tool for averting risk factors associated with NCDs in the upcoming years [21]. Enabling resources in resource-constrained LMICs, such as mobile-based health technologies, can help strengthen health system response to NCDs by offering flexible ways for communities and healthcare professionals to receive healthcare services. With regard to the considerable attention that mHealth has received globally and in LMICs, this debate highlight factors that determine health systems readiness for adoption of mHealth technology to address NCDs in the context of LMICs. There are some limitations concerning the rollout of mHealth intervention for NCDs in resource-constrained settings. In LMICs, much of the focus is on small pilot mHealth projects, which are rarely followed-up and taken to a large scale for widespread implementation. The lack of rigorous evaluation of the pilot mHealth studies threatens the trustworthiness of the mHealth field. To adequately understand the impact of mHealth interventions, it is vital to undertake more rigorous evaluations for the successful pilot mHealth initiatives that have potentials to be scaled-up [18,30]. Currently, there is insufficient empirical and policy evidence on this research area. Future research and policy dialogue should be directed to explore the health systems preparedness and willingness for mHealth adoption principally to address NCDs in the context of LMICs. There is an eminent need to bring together variety of stakeholders for strengthening health systems to address NCD burden in LMICs through mHealth.

## Paper context

While mHealth field offers a great promise to prevent and control NCDs in LMICs: there is a great debate going on to explore health systems readiness for adopting mHealth technology to address NCDs in LMICs. The health systems readiness for mHealth is determined by various factors including willingness of institutions, healthcare providers and communities to adopt mHealth. To integrate mHealth into existing health systems, these factors should be dealt up-front to improve health systems response for NCDs.
